# Modelling of diabetes knowledge, attitudes, self-management, and quality of life: a cross-sectional study with an Australian sample

**DOI:** 10.1186/s12955-015-0303-8

**Published:** 2015-08-19

**Authors:** Yee Cheng Kueh, Tony Morris, Erika Borkoles, Himanshu Shee

**Affiliations:** College of Sport and Exercise Science, Victoria University, Melbourne, Australia; Unit of Biostatistics and Research Methodology, School of Medical Sciences, Universiti Sains Malaysia, Kelantan, Malaysia; College of Business, Victoria University, Melbourne, Australia

**Keywords:** Knowledge, Attitudes, Self-management, Quality of life, Type 2 diabetes

## Abstract

**Background:**

Quality of life (QoL) is an important aspect of wellbeing for people with chronic conditions like type 2 diabetes, making it a noteworthy outcome. Knowledge about diabetes, attitudes, and self-management of diabetes are key factors that might directly or indirectly impact QoL. However, little is known about the inter-relationships between diabetes knowledge, attitudes, self-management and QoL among people with type 2 diabetes mellitus (T2DM). The aim of this study was to examine a model describing the relationship between diabetes knowledge, attitudes, self-management, and QoL of people with T2DM that is based on previous research linking pairs of these variables.

**Methods:**

A cross-sectional study design was employed in this research. A total of 291 participants, 192 males and 99 females, with T2DM, whose mean age was 55.8 (standard deviation = 11.09) completed questionnaires measuring diabetes knowledge (Diabetes Knowledge Scale), attitudes (Diabetes Integration Scale -19), self-management (Summary of Diabetes Self-care Activities Scale), including the aspects of diet, exercise, blood glucose testing, and foot care, and QoL (Diabetes Quality of Life Scale), comprising the aspects of satisfaction and impact on QoL respectively. To examine the model we proposed relating these variables, data were analysed using the path analysis.

**Results:**

In the final model, diabetes knowledge was a significant predictor for attitudes and self-management in terms of blood glucose testing. Attitudes was a significant predictor for self-management in terms of diet. In addition, self-management in terms of blood glucose testing was a significant predictor of impact of QoL, and self-management in terms of diet was a significant predictor of satisfaction and impact of QoL. Self-management in terms of exercise was a significant predictor of satisfaction in QoL. The final model reflected a good fit (*χ*^2^ (14) = 22.52, *p* = 0.069; CFI = 0.983; GFI = 0.983; RMSEA = 0.046).

**Conclusions:**

Diabetes knowledge, attitudes, and self-management are important factors that can impact the QoL among people with type 2 diabetes.

## Background

Diabetes is a major progressive and life-threatening disease with many complications. It is likely that people with diabetes will experience lower levels of quality of life (QoL) as the disease progresses. This will affect their motivation to maintain their health. Researchers have suggested that people with diabetes experience a decrease in their QoL compared to healthy individuals [1]. A number of studies have shown the association between diabetes and QoL [2, 3]. This is not surprising because diabetes affects many important aspects of physical health, such as vision, sensation in the extremities, kidney function, diet, and the capacity to carry out activities of daily living [4–6]. Diabetes progressively demands the dedication of more time to its management, including taking oral medication, self-administering insulin injections, and testing blood sugar level several times a day. Diabetes can also lead to loss of personal income and productivity due to restrictions in the amount and type of work that people can perform, as well as early retirements due to diabetes-related complications [7–9].

Some personal lifestyle factors are associated with improved health in terms of QoL among people with diabetes. The combination of reduced fat and sugar in the diet and increased exercise have not only been shown to improve glycosylated haemoglobin measures, which indicate positive control of blood glucose levels among people with diabetes, but these lifestyle changes also improve general QoL significantly [10]. Therefore, self-management is an important part of daily life for people with diabetes. It has been reported that approximately 95 % of diabetes care is self-treatment or self-management [11]. To control diabetes, individuals must monitor their daily lifestyle behaviour and often they must change long-held habits.

Self-management activities demand a great effort, which many people find difficult to incorporate into their daily life [12, 13]. Health professionals have to recognize that long-term behaviours are very hard to adjust or change. Thus, understanding factors that are associated with individual diabetes self-management behaviours is important for health professionals. Attitudes of people with diabetes can play an important role in their emotional response, as well as affecting their efforts to manage their diabetes in everyday life. Researchers have proposed that individuals who have positive attitudes toward managing their diabetes will be more likely to adjust their self-care behaviour in order to control their blood glucose levels than those who have negative attitudes [14, 15].

In addition, knowledge of diabetes can become a cornerstone in decision making on diet, exercise, blood glucose monitoring, use of medication, weight control, and foot care [16]. In a study to determine the management behaviour of people with diabetes, Kamel et al. observed a linear relationship between overall knowledge level and diabetes management [17]. People with diabetes lacked knowledge and consequently had low levels of self-care practices. This is expected, as specific health information may be necessary before personal health action is carried out. Therefore, it is important to consider how knowledge of diabetes and attitudes of people with diabetes can impact their self-management practices and also their QoL.

The limited research that has been conducted typically examined the relationship between two variables associated with diabetes, including knowledge and attitudes [18], knowledge and self-management [16, 17, 19, 20], attitudes and self-management [21, 22], and self-management and QoL [3]. In extensive literature searches, we did not identify any previous research that has examined the relationships between diabetes knowledge, attitudes, self-management, and QoL among people with type 2 diabetes (T2DM) in a single study. Most researchers examined bivariate relationships between knowledge, attitudes, self-management and QoL. A number of issues can be explored by examining all the relationships together in one model, in which the predicted paths between the key variables are examined based on the paths suggested by previous research on bivariate relationships. Examination of all these paths at the same time can be achieved by using a structural equation modelling approach. Other factors such as age and duration of diabetes should also be considered when testing the relationship between knowledge, attitudes, self-management and QoL. This is because the age of people with T2DM diabetes had been showed to have a relationship with diabetes knowledge [19, 23]. Researchers have reported that duration of diabetes was associated with level of diabetes knowledge [20]. It has also been found that people with shorter diabetes duration were more concerned about their management of diabetes than those with longer duration that is, they had more positive attitudes to living with diabetes [24].

**Fig. 1:**
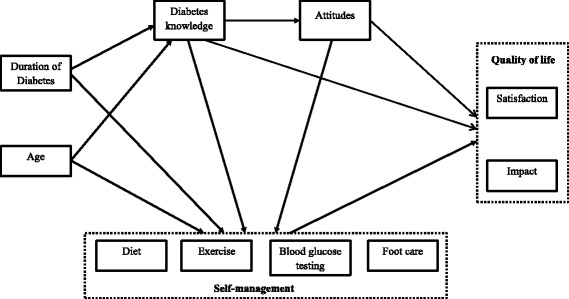
Hypothesized structural model. Hypothesised structural model of age, duration of diabetes, diabetes knowledge, attitudes, self-management, and QoL in people with type 2 diabetes

In the present study, we aim to address the gap in research on the inter-relationships between the key variables of knowledge, attitudes, self-management and QoL by testing a model describing these inter-relationships, as well as considering the influence of age and duration of diabetes on the relationships, among people with T2DM. Based on the review of the literature, we hypothesized that knowledge is associated with more positive attitudes, knowledge and attitudes are associated with higher levels self-management, and, in turn, more effective self-management is related to higher levels of QoL. For the variables of age and duration of diabetes, we hypothesized positive associations with knowledge and self-management. This model is depicted in Fig. 1.

## Methods

### Procedure

We conducted a cross-sectional study, in which we distributed self-administered questionnaires measuring the key variables identified in the review of literature at two hospitals (The Alfred Hospital and the Western Hospital) in Melbourne, Australia. Due to the large sample size required in path analysis and time constraints in data collection, we used a convenience sampling method to recruit the participants. Potential participants with T2DM were invited to participate in the study during their visits to the hospital for clinical appointments with their physician or diabetes educator in the outpatient clinic. The questionnaire packs were also sent out to people with T2DM who were identified through the patient database in the hospital and had not been invited to participate in the study at the outpatient clinic. Participants took approximately 30 min to complete the demographic form and the four questionnaires and return the completed questionnaires to the researcher. Participants for this study were males and females, aged over 18 years. We only included individuals who were diagnosed with T2DM by medical practitioners for at least a year and were registered with the specific hospitals. They had to possess sufficient knowledge in English to be able to read, understand, and answer the items in the four questionnaires. The questionnaire packs were distributed to the participants through the hospitals. We obtained 291 usable sets of responses by the end of the study. Participants completed an informed consent process. No monetary reimbursement was given to the participants. All participants were volunteers in the present study. This study was approved by the Human Research Ethics Committee of the Alfred Hospital, Western Hospital, and Victoria University, Melbourne, Australia.

### Participants

Participants were 291 adults with T2DM (192 males, 99 females). Participants’ ages ranged from 21 to 70 years, with mean age of 55.8 years (*SD* = 11.09 years). Participants’ time since diagnosis with T2DM (duration of diabetes since diagnosis) ranged from 1 to 39 years, with a mean of 11.9 years (*SD* = 9.01 years). Approximately half (53.6 %) of the participants, had been treated with insulin at the time of data collection. The majority of the participants, in this study, 74.6 %, had completed at least high school education.

### Instruments

The measures included a demographic form, including health treatment questions, and four questionnaires, measuring diabetes knowledge, attitudes to living with diabetes, self-management, and diabetes QoL.

The demographic form included questions about gender, age, duration of diabetes since diagnosed, education level, employment status, and type of diabetes treatment.

The Diabetes Knowledge (DKN) scale was developed in the early 1980s. The developers reported that the DKN has strong psychometric criteria of reliability and validity, which meet the need for a brief measure of diabetes knowledge [25]. The DKN scale has been used with samples of participants ranging in age from teenagers to the elderly, and with individuals from a variety of ethnic backgrounds. It is designed to be self-administered by respondents [26]. The DKN questionnaire contains 15 multiple-choice items each related to a basic aspect of diabetes or its treatment. A score of 1 is assigned for a correct response and 0 for an incorrect response. The total score is calculated by summing the scores from the 15 items. The total scores are then converted to percentages. Higher scores on this measure indicate a higher level of diabetes knowledge. The DKN is a reliable measure of diabetes knowledge for researchers investigating the relationships between knowledge, psychological and social factors, health status, and metabolic control [26]. Although the DKN was developed in 1980s, it is still widely used by researchers across different countries (e.g., [27–30]).

The Diabetes Integration Scale-19 (ATT19) is a short version of the ATT39, measuring psychological adjustment and attitudes toward diabetes using a 19-item self-report questionnaire [31]. There are 19 self-report attitudinal items. Participants rate their agreement or disagreement with each item on a 5-point Likert scale ranging from 1 (*I disagree completely*) to 5 (*I agree completely*). Sixteen items are reverse scored (i.e., items 1, 2, 3, 4, 5, 6, 7, 8, 9, 10, 12, 13, 14, 16, 17, and 19), so a high score on these items reflects a positive attitude toward having diabetes and better adjustment compared to lower scores. The aggregate score of ATT19 was obtained by adding up the scores from all the 19 items. The internal consistency of the ATT19 is alpha = 0.84 [31]. The ATT19 has been broadly utilised by researchers in measuring attitude of people to having diabetes as a reflection of psychological adjustment to living with the condition (e.g., [30, 32–36]).

The Summary of Diabetes Self-Care Activities (SDSCA) is a brief, 7-day, self-report measure of the frequency of conducting a number of key diabetes self-care activities [37]. The SDSCA consists of 11 core items assessing self-management behaviour in diet, exercise, blood glucose testing, foot care, and smoking status. Average scores are calculated for each of the four areas assessed by the SDSCA. The average inter-item correlations of the SDSCA subscales generally exceeded 0.5 and were considered to be high [38]. The SDSCA self-administered questionnaire was also considered to be the most clinical practical and cost-effective approach to self-care assessment [37]. Researchers have frequently used the SDSCA questionnaire, particularly in measuring diabetes self-management behaviours among people with T2DM (e.g., [39–43]).

The Diabetes Quality of Life questionnaire (DQoL) was developed in the early 1980s and was intended to evaluate the relative burden of an intensive diabetes treatment regimen in the Diabetes Control and Complications Trial (DCCT) [44]. The DCCT Research Group contributed their expertise in the development of this measure and the items were derived from the literature on psychosocial aspects of diabetes, as well as from input from patients and clinicians, diabetologists, diabetes educators, nurses, and mental health professionals familiar with diabetes then, repeatedly reviewed the DQoL measure [44]. The DQoL questionnaire used in this study contains 35 self-report items with 15 items measuring satisfaction, 20 items measuring impact. Answers are given on a 5-point Likert scale rated from 1 (*very satisfied, no impact*) to 5 (*very dissatisfied, very impacted*). High scores on the satisfaction scale reflect high levels of satisfaction related to treatment, self-management, social, and physical functioning and, thus reflect high QoL. High scores on the impact scale indicate high levels of impact related to adverse diabetes events, restrictions to social and physical functioning and, thus reflect low QoL. The total score is calculated by summing the scores from each of the items for satisfaction and items for impact and then converting the total to a percentage. The DQoL is a reliable and valid tool in measuring diabetes QoL. It has high test-retest correlations in the 0.78 to 0.92 range in both adults and adolescents with diabetes [45]. In the past 10 years, the DQoL scale has been translated into different languages and used by researchers in different countries, including Hong Kong (Chinese version) [46], Taiwan (Chinese version) [47], Thailand (Thai version) [48], and Turkey (Turkish version) [49].

### Statistical analyses

The data was entered into the Statistical Package for the Social Sciences (SPSS) version 20.0. In the data set, there were more male than female participants. Therefore, preliminary analysis on gender differences were first carried out using independent *t*-test on all study variables. Then, the descriptive statistics was used to describe the study variables. Correlations were used to explore the strength of the relationship between the study variables. Duration of diabetes since diagnosis, age of participants, diabetes knowledge, attitudes, and four aspects of self-management (diet, exercise, blood glucose testing, and foot care), and two subscales of DQoL (satisfaction and impact) were examined.

A Parsimonious model is aimed at and preferred in path analysis [50]. Therefore, the significant correlations of the variables were taken into consideration when the initial hypothesed path model was developed. Using structural equation modelling (SEM) techniques, path analyses were conducted using the Analysis of Moment Structures (AMOS) version 17.0 software. Several fit indices were considered to determine the goodness-of-fit of the path model. The statistics included chi-squared statistics, with a desired value of *p* > 0.05, the root mean square error of approximation (RMSEA), with a desired value of less than 0.05, the comparative fit index (CFI) and goodness of fit index (GFI) with desired values of greater than 0.95 [51]. After obtaining the final path model, the significance of the indirect effect of diabetes knowledge on QoL was examined through other variables. This was done by requesting AMOS to determine the significance level (*p*-value) of the indirect effects by performing bootstrapping in the modelling analysis [51].

## Results

### Characteristics of participants

The gender of participants in this study included more males (*n* = 192, 66 %) than females (*n* = 99, 34 %). Nevertheless, the preliminary analyses on gender differences on all study variables indicated that there were no statistically significant gender differences on any of the study variables included in the study. Therefore, all data was used for subsequent analyses. Other descriptive statistics are presented in Table 1. On average, the participants scored above the midpoint on all measures except for impact of DQoL scale. For the impact of DQoL scale, the low score on impact indicates a relatively high level of diabetes QoL. Thus participants generally experienced high satisfaction for and low impact of QoL, that is, they reported positive QoL. Participants also generally scored higher in terms of the self-management of diet and blood glucose testing compared to exercise and foot care (see Table 1).

**Table 1:** Participants characteristics (*n* = 291)

	Mean (SD)	Potential range (midpoint)	Number of participant (percentage)
Age (years)	55.8 (11.09)		
Duration of Diabetes since diagnosis (in years)	11.9 (9.01)		
Education background:			
Less than high school			74 (25.4 %)
High school			111 (38.2 %)
College			46 (15.8 %)
University			60 (20.6 %)
Type of treatments:			
Diet			25 (8.6 %)
Diet & tablet			110 (37.8 %)
Diet & Insulin			84 (28.9 %)
Diet, tablet, & insulin			72 (24.7 %)
Diabetes knowledge	61.7 (19.61)	0 – 100 (50)	
Attitudes	63.4 (11.68)	19-95 (57)	
Self-management:			
Diet	4.8 (1.56)	0-7 (3.5)	
Exercise	3.5 (2.26)	0-7 (3.5)	
Blood glucose testing	4.9 (2.49)	0-7 (3.5)	
Foot care	3.4 (2.58)	0-7 (3.5)	
Quality of life:			
Satisfaction	65.3 (17.60)	0-100 (50)	
Impact	28.5 (14.32)	0-100 (50)	

### Relationship between variables

Correlations between the study variables are presented in Table 2. The significant correlation coefficients range from *r* = −.12 to *r* = −.59. The strongest correlations were shown between satisfaction and impact, which measure QoL of T2DM. The higher was the levels of satisfaction of individuals in regard to their QoL, the lower was the impact of QoL. Strong correlations were also shown between attitudes and impact of QoL and between attitudes and satisfaction with QoL. A moderate correlation was also shown between self-management of diet and satisfaction with QoL. There were significant negative correlations between age and diabetes knowledge, and duration of diabetes since diagnosis and self-management of exercise.

**Table 2:** Correlation of study variables (*n* = 291)

Variable	1	2	3	4	5	6	7	8	9	10
1. Age	1	.294**	−.155**	.089	.156**	−.064	.153**	.170**	.181**	−.067
2. Duration of diabetes since diagnosis (in years)		1	.205**	−.055	.048	−.119*	.205**	.173**	.001	.115
3. Diabetes knowledge			1	.252**	.083	.090	.154**	.095	.001	−.006
4. Attitudes				1	.233**	.091	.107	.020	.503**	−.503**
5. SM-Diet					1	.290**	.380**	.248**	.419**	−.216**
6. SM-Exercise						1	.023	.111	.307**	−.134*
7. SM-Blood glucose testing							1	.211**	.201**	.018
8. SM-Foot care								1	.128*	.027
9. Quality of life – Satisfaction									1	−.590**
10. Quality of life - Impact										1

*SM* self-management

*p < .05

**p < .01

### Structural model

**Fig. 2:**
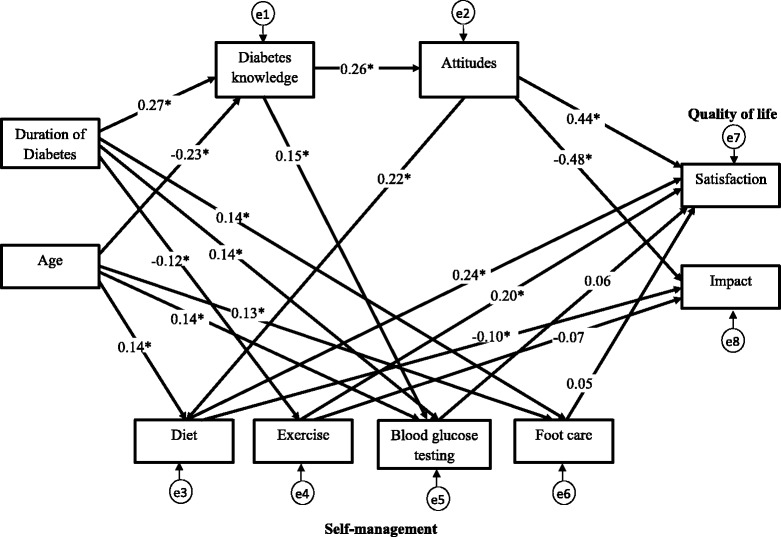
Initial structural model. Initial structural model of age, duration of diabetes, diabetes knowledge, attitudes, self-management, and QoL in people with type 2 diabetes. Path loadings are standardized path coefficient. *p < 0.05

The hypothesized relationships based on the empirical findings from previous research were illustrated in Fig. 1. They were reviewed in the Background section and then summarized at the end of that section. Due to the multidimensional nature of self-management, the different aspects of self-management are assessed separately [37, 38]. Therefore, in this study the four aspects of self-management, namely diet, exercise, blood glucose testing, and foot care, were treated as distinct variables in the model. Based on the literature review and results from the correlation analysis in this study, the initial hypothesized model was developed and illustrated in Fig. 2. The initial hypothesized model did not result in a good fit to the data (*χ*^2^ (26) = 229.49, *p* < 0.001; RMSEA = 0.164; CFI = 0.612; GFI = 0.867). In evaluation of each of the 19 path relationships in the initial hypothesized model, some paths were not significant and one variable, foot care contributes to the poor fit of the model. After considering the results of the initial hypothesized model and theoretical issues, some modifications were made. Path relationships that were not significant were removed and variable that contributed poor fit of the model was omitted, and some additional path relationships were added to the model. The non-significant paths which were removed were pathways linking exercise to impact, blood glucose testing to satisfaction, and foot care to satisfaction in QoL of T2DM. Adequate theoretical support was identified to investigate the new path relationships suggested by the modification index. These paths were added into the model one at a time and the model was re-tested each time a new path was added. Any variable that did not contribute as a significant predictor was also removed from Model 2. Thus, foot care was removed from the model as it did not contribute as a predictor for DQoL variables. Arrows that represent correlations between duration of diabetes since diagnosis and age, satisfaction and impact, diet and exercise, diet and blood glucose testing were introduced to the final path model. The fit indices for the final model resulted in good fit (*χ*^2^ (14) = 22.52, *p* = 0.069; RMSEA = 0.046; CFI = 0.983; GFI = 0.983). The regression coefficients between the variables were improved and all paths were significant and theoretically important.

**Fig. 3:**
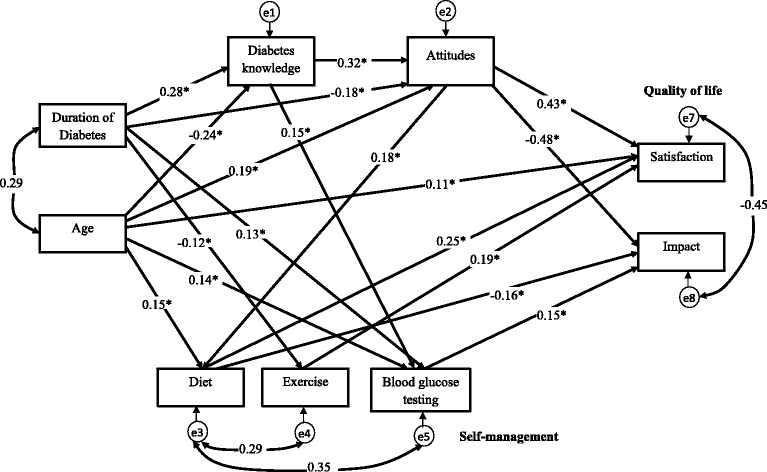
Final structural model. Final structural model of age, duration of diabetes, diabetes knowledge, attitudes, self-management, and QoL in people with type 2 diabetes. Path loadings are standardized path coefficient. *p < 0.05

In the final model as depicted in Fig. 3, more positive attitudes and greater self-management of diet directly enhance satisfaction and reduce the impact of QoL. Self-management in terms of more frequent blood glucose testing directly affects the impact of QoL, increasing impact, whereas higher levels of exercise directly increase satisfaction of QoL. Besides, attitudes was significant affects diet, with higher level of positive attitudes directly increase the self-management in terms of diet. Knowledge was significant affects attitudes and blood glucose testing, with higher level of knowledge directly increase the level of positive attiudes and more frequent in self-management in terms of blood glucose testing.

Knowledge did not directly affect diabetes QoL. Further analysis was conducted to examine the indirect relationship between knowledge and diabetes QoL. The result shows that knowledge affects satisfaction of diabetes QoL indirectly through attitudes (*p* = 0.011) and affects impact of diabetes QoL indirectly through self-management in terms of blood glucose testing (*p* = 0.007).

## Discussion

The present study provides valuable insight into the use of path analysis which is an extension of multiple regressions for testing the hypotheses in data related to diabetes knowledge, attitudes, self-management, and QoL among people with T2DM. It is different from other traditional regression analysis where only one hypothesis and dependent variable could be tested. Path analysis which is the family of SEM, allows for exploration of the web of relationships between levels of diabetes knowledge, attitudes, self-management, QoL, and extraneous variables, such as age and duration of diabetes, among people with T2DM in one single model. In other words, it tests multiple hypotheses in one single path model which was the highlight of the present study.

In this study, we identified a significant relationship between diabetes knowledge and blood glucose testing. This yields an important insight for health care providers, which indicates that we should continue to educate people with T2DM about the effects of diabetes and how to manage their diabetes to minimise its impact. To achieve this it is necessary to find additional ways to increase the knowledge that people have about their diabetes and especially about self-management. By increasing their diabetes knowledge, people with T2DM may learn to maintain good control of their blood glucose level, which can prevent other complications related to diabetes. Although, there was no direct significant relationship between diabetes knowledge and QoL in the present study, we found that knowledge affected QoL indirectly through attitudes to living with diabetes and self-management in terms of blood glucose testing. Although, some researchers had reported the non-significant result [3, 52] between knowledge and QoL, the present study gave a different insight that knowledge about diabetes did not directly impact on QoL but indirectly impact QoL through other indicators such as attitudes and self-management in terms of blood glucose testing.

The final path model indicates that more positive attitudes led to more regular diet self-management. There is a possibility that attitudes can be affected by certain symptoms of depression, influencing self-management behaviours, such as diet. Researchers have shown that symptoms of depression were related to negative attitudes and health behaviour, including poorer self-efficacy for diet [53]. Thus, it is important to create more positive attitudes to managing diet, which will then lead to more effective actual management of diet. The present results indicate that people with T2DM who had more positive attitudes to living with diabetes were more likely to have higher levels of QoL than those who had negative attitudes. This supports the results found by Menard et al. in a randomized controlled trial study, where after 12 months of intervention, a positive relationship between attitudes and QoL was observed among the participants with T2DM [3]. This provides important insight for health care providers that enhancing positive attitudes among people withT2DM has the potential to increase their QoL for living with diabetes.

In the final model, we excluded the variable foot care, which is part of self-management, and this improved the fit of model. Among the four components of self-management practices, foot care had the lowest mean (3.38; being practised on average 3 times over the past 7 days) for self-reported self-management. This indicates that, in general, participants reported practising the other aspects of self-management more regularly than foot care to control their illness and its impact. This may be because little education is provided to people with T2DM regarding foot self-care, particularly for those people who are considered at low risk of developing diabetes-related foot complications [54]. It is widely acknowledged that proper foot self-care is not carried out by the majority of people with T2DM [55]. Further research should be conducted to understand what influences the relatively low level of attention paid to foot-care by people with T2DM, so that foot care awareness among people with T2DM can be increased through appropriate education and interventions implemented by appropriate health care providers.

The final model in the present study indicated that more regular diet self-management led to higher levels of satisfaction with treatment and lower levels of impact of diabetes, which both reflect more positive QoL. In this study, it also showed that more regular self-management of exercise led to higher levels of satisfaction with treatment in QoL. In another study using regression analysis, Smith and McFall also reported that exercise was associated with improved QoL among people with diabetes [56]. However, there was no significant path relationship between exercise and impact of QoL in the present study. People with health problems associated with T2DM, such as foot pain and injury, may face challenges adhering to daily exercise routines. Thomas et al. reported that some people with T2DM worried that their exercise could lead to deterioration in their health and create unpleasant feelings. Thus, people with T2DM may perceive that exercising does not have an important impact on their QoL. Moreover, people with diabetes have been found to undertake less physical activity than people without diabetes [57]. Hence, people with T2DM who exercise regularly to improve their health may experience more satisfaction than those with T2DM who do not do as much exercise because they are able to do exercise regardless of their diabetes condition.

This study also yielded interesting findings that more regular self-management in terms of blood glucose testing was associated with higher impact of diabetes among people with T2DM. These results support the study by Franciosi et al. who found that self-monitoring of blood glucose is associated with psychological burden among people with T2DM [58]. They explained that self-monitoring blood glucose more than one time per day was significantly related to higher levels of distress, worries, and depressive symptoms. This may explain the positive relationship between blood glucose testing and impact of QoL found in the present study.

During the validation of a new knowledge scale for people with T2DM, Rothman et al. found that a higher level of knowledge about diabetes was positively correlated with duration of diabetes, which was also found in the present study [59]. In other words, people with greater knowledge about diabetes had been diagnosed with T2DM for longer, so they had more time to accrue knowledge from a variety of sources, such as medical practitioners, clinics, diabetes support organisations, and health literature. In the present study, longer duration of diabetes since diagnosis was associated with more regular self-management in terms of blood glucose testing, but the reverse was found to be the case for self-management in exercise. This yielded the interesting finding that people with longer duration of diabetes since diagnosis were less likely to participate in exercise regularly than those who had shorter duration of diabetes since diagnosis. In terms of age, the present results are parallel with other studies where younger age predicted greater diabetes knowledge [17, 60].

In addition, the path analysis revealed that age and duration of diabetes since diagnosis were significant predictors of diabetes knowledge. Those who were older were reported to have lower level of diabetes knowledge than those who were younger. However, those who had longer duration of diabetes since diagnosis were reported to have higher level of diabetes knowledge than those who had shorter duration of diabetes. It is reasonable to expect that longer duration of T2DM would be associated with greater age. This is also shown in the final path model where duration of diabetes since diagnosis was positively correlated with age. This has the appearance of a paradox. However, examination of the scatter plot for age and duration independently from other variables, indicated that the correlation is small. Similarly, the scatter plots for age and knowledge and for duration and age reflect small correlations with almost rectangular distributions of points. Thus, the opposite directions of these small correlations should be treated with caution.

Several limitations to this study should be noted. Cross-sectional design was used in this study. Causal relationships between diabetes QoL and other variables cannot be assumed. In addition, the sampling method was based on convenience sampling and participants were volunteers. Thus, it is unclear to what extent these results can be generalised to other people especially those with severe diabetes complications who could not participate in the current self-report research. It is important to clarify that the sample in this study is generally well-adjusted of people with T2DM, who are treated as outpatients in the hospitals. Thus, conclusions about the study need to be carefully drawn and limited to a population defined by these characteristics.

### Further research

In the present study, we did not measure the number of complications that participants had, which research has shown can affect their QoL [61–63]. Participation in this research was based on volunteering. Therefore, people with serious complications may or may not have participated in this research by choice or circumstances. For example, in the present study, people with T2DM and with serious or multiple complications, could be forced to stay at home, so they may not have been able to complete the questionnaires and send them back to the researchers. Those who had serious and multiple complications and were not able to participate in the present study may show different relationships between the key variables in this study. Two questions to be considered in further research arise from this observation. First, it is important to know whether people with T2DM with multiple complications have less diabetes knowledge, more negative attitudes to T2DM, less regular self-management of T2DM, and lower levels of QoL of T2DM than those who do not have complications. For example, individuals with T2DM who have also suffered other complications, such as heart disease, stroke, peripheral vascular disease, and vision disorders may experience more negative attitudes to T2DM and lower levels of QoL of T2DM. It would be interesting to examine how diabetes related complications affect the study variables in this research. Second, it would be interesting to examine whether the same pathway relationships between diabetes knowledge, attitudes to T2DM, self-management of T2DM, and QoL of T2DM arise for those who do have multiple diabetes complications. Outcomes from such research will have implications for ways to improve the self-management of T2DM and increase the level of QoL of T2DM among people with T2DM and complications or comorbidities.

Another perspective from which to consider the relationship of QoL with other variables in the present research is reversing the direction of the relationships. QoL could be of direct importance to the self-management of T2DM. It is possible that people with higher levels of QoL will have greater motivation to learn more about T2DM, thus, this should increase their knowledge. Having higher QoL could also enhance the attitudes of people with T2DM. This could occur because high QoL encourages people to seek further knowledge and increased knowledge leads to more positive attitudes, as suggested in the path analysis in the present research. Additionally, QoL could directly impact attitudes because having higher QoL gives people a more positive view of life, including the way they feel about coping with their diabetes. People with more positive experience of well-being and higher QoL could possibly do more self-management activities to maintain their health status or limit the negative physical impact of diabetes. Thus, researchers could examine the relationship between QoL and the other key variables both by reversing the paths in a cross-sectional model similar to the present model, and through longitudinal data analysis. This can be achieved by following up participants over time with repeated measures of each variable of interest. Therefore, the effect of QoL on knowledge, attitudes, and self-management could be examined more thoroughly. As measures are taken at different times, interventions could be added to examine the changes of these variables prospectively, which would add more value to the two studies suggested above. In such analyses, it is would be useful to examine the role of variables such as duration of diabetes since diagnosis ans severity of diabetes as moderator variables. For example, although we found that greater knowledge about diabetes predicted more frequent glucose testing, individuals with good control of their blood glucose, that is, lower severity, might not need to test it so regularly, although their level of control might result from superior knowledge.

## Conclusions

In the present study, the final model revealed that practising self-management more regularly in relation to diet, exercise, and having more positive attitudes led to higher levels of satisfaction in QoL among people with T2DM. Similarly, practising diet self-management more regularly, and having more positive attitudes led to lower levels of negative impact of QoL among people with T2DM. Improving self-management in diet, exercise and encouraging more positive attitudes to living with diabetes should improve QoL among people with T2DM in Australia. Ensuring people with T2DM have high QoL is a laudable goal in its own right and is consistent with our conviction that people with T2DM should aim for the highest QoL they can achieve.
